# Photochemistry with laser radiation in condensed phase using miniaturized photoreactors

**DOI:** 10.3762/bjoc.8.135

**Published:** 2012-07-31

**Authors:** Elke Bremus-Köbberling, Arnold Gillner, Frank Avemaria, Céline Réthoré, Stefan Bräse

**Affiliations:** 1 Fraunhofer Institute for Laser Technology, Steinbachstrasse 15, D-52074 Aachen, Germany; 2 Institute of Organic Chemistry, Karlsruhe Institute of Technology, Fritz-Haber-Weg 6, D-76131 Karlsruhe, Germany; 3 Institute of Toxicology and Genetics, Karlsruhe Institute of Technology, Hermann-von-Helmholtz-Platz 1, D-76344 Eggenstein-Leopoldshafen, Germany

**Keywords:** azides, chemical diversity, flow chemistry, heterocycles, laser, micro reactor

## Abstract

Miniaturized microreactors enable photochemistry with laser irradiation in flow mode to convert azidobiphenyl into carbazole with high efficiency.

## Introduction

Classical combinatorial chemistry [1–2] approaches usually aim at the synthesis of multi-milligram amounts of new compounds to extend screening decks used in multiple screening campaigns [3]. An alternative method enabled by the maturing microreaction technology and the use of flow chemistry [4–6] is the integration of synthesis and screening in one integrated lab-on-a-chip approach [7].

Using this methodology we have integrated photochemistry in a miniaturized reaction setup to enable combinatorial flow chemistry in lab-on-a-chip applications.

Photochemical processes are in this case particularly interesting because of their enhanced molecular activation [8]. Photochemistry in microreactors is an emerging research area [9], and especially photocatalytic reactions have been investigated in detail by Matsushita et al. [10–11]. To date there are a couple of reported examples combining miniaturized reaction systems with synthetic laser photochemistry [5–6,9,12–28].

The influence of photons, which are delivered via a suitable light-transparent window, on the processes running in miniaturized photoreactors, is investigated with a focus on increasing the yield and selectivity as well as decreasing the reaction time. Photochemistry with laser radiation is a promising tool to broaden the application spectrum of miniaturized systems, by facilitating a powerful activation step due to a wide range of available wavelengths and energy ranges [29–30]. Moreover, the optical systems can be designed in a way that the reaction initiation by photons and an additional online analysis of the running reaction is feasible.

## Results and Discussion

### Design and fabrication

In order to realize photochemical synthesis, several reactors and small reactor arrays with reaction volumes of approximately 1 mL down to 35 µL were developed. These reactors were especially designed for the stimulation of photochemical reactions (UV–vis radiation) as well as for demanding reaction conditions, such as the rapid elevation of temperature (with pulsed IR-laser radiation) or pressure pulses (due to the evaporation of the solvent upon the introduction of energy).

Several microstructured reactor types were designed and produced for reactions in the liquid phase. They are equipped with quartz-glass cover plates, transparent to the laser radiation, pressed onto an appropriate sealing material. Moreover, channels suitable for the mixing and reaction of two or more isopycnic solutions were built in a polymer bloc by mechanical treatment [31]. The provision of bubble-free fluid is ensured in this case by microchannels in at least two levels, which are built from corresponding structured layers. These reactors were made of polyether ether ketone (PEEK) and polytetrafluoroethylene (PTFE) [32] to study the influence of side reactions with the reactor material, which could reduce the yield of the desired reaction product. The multilayer system is placed in a stainless-steel frame.

**Figure 1:**
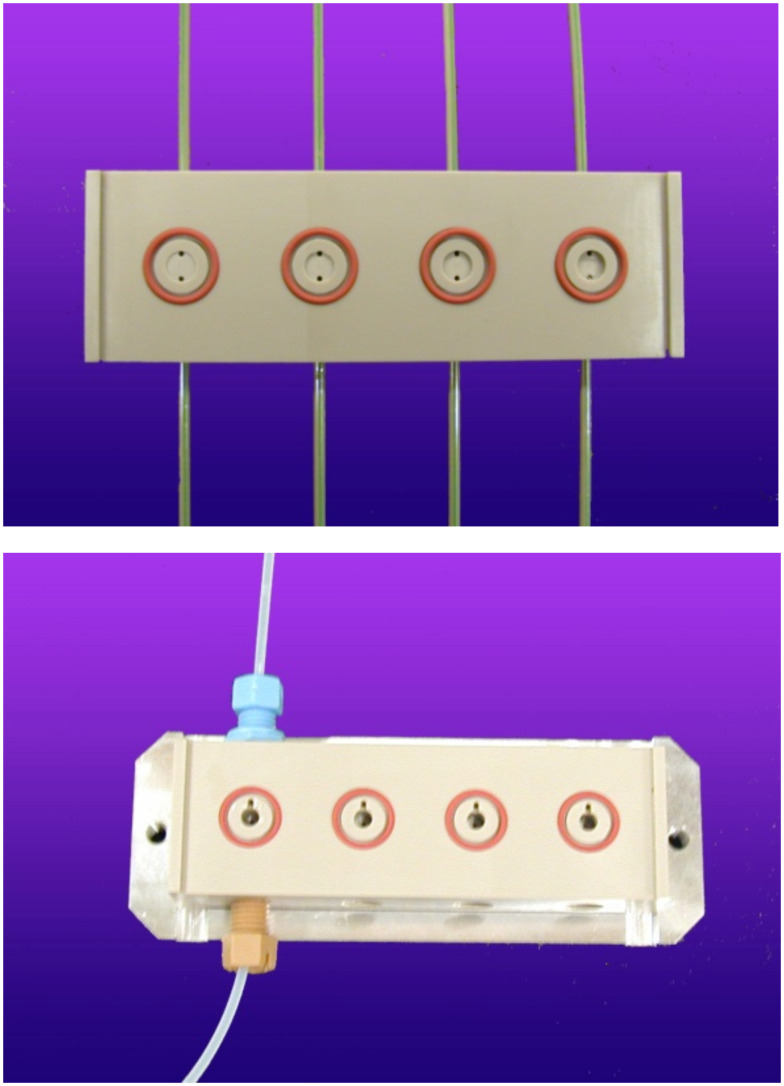
Four-fold PEEK-reactors with increasing chamber depths from left to right and different techniques of fluid connection; top: glued PEEK-capillaries, bottom: ¼ in. screw connections.

With this type of reactor, it is possible to realize a series of reactions in parallel by arranging the reactor chambers in an (n × m) matrix. The microreactors applied for this study have four reaction chambers with varying volumes of the chambers due to increasing depth, and different connections for the reagent entrance (Figure 1).

### Photochemistry

**Scheme 1:**
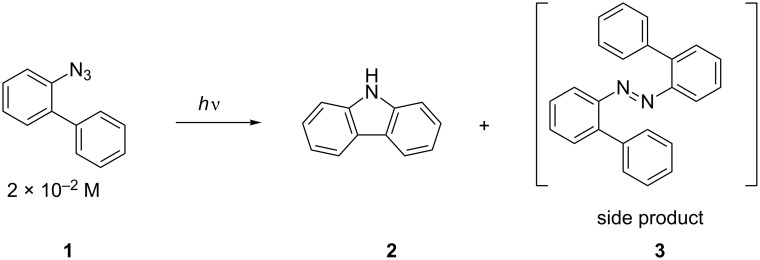
Synthesis of carbazole (**2**) by photolysis.

The combinatorial synthesis of heterocycles and among them of carbazole is of particular interest since they are potential active pharmaceutical compounds [33–35]. The photolysis of 2-azidobiphenyl (**1**) with the help of a conventional UV-lamp has been used for the synthesis of carbazole (**2**) since 1960 (Scheme 1) [36–39].

**Scheme 2:**
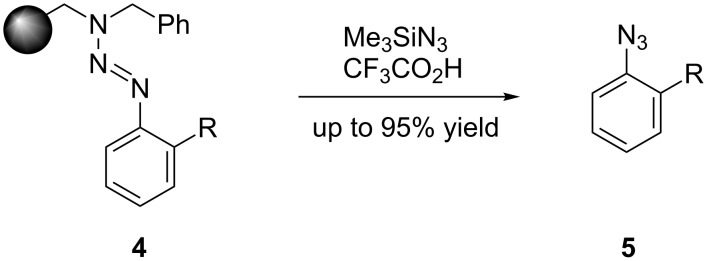
Synthesis of arylazides **2** by solid-phase synthesis.

However, there are only a few examples of substituted products obtained by this reaction, and several side-products, such as the corresponding azo-derivatives, are usually formed [36,40–44]. During the past few years, we successfully employed triazene resins, such as **4**, which are readily available from aniline in the synthesis of a library of aromatic derivatives [45–48]. Moreover, triazene-resins are perfectly suitable for the synthesis of arylazides **5** (Scheme 2) [49].

The photochemical decomposition of arylazides into carbazoles is appropriate for application in miniaturized photoreactors, since significant results can be observed by an online analysis through HPLC and GC [50]. Because of the miniaturization, online analysis is especially suitable for our setup.

**Figure 2:**
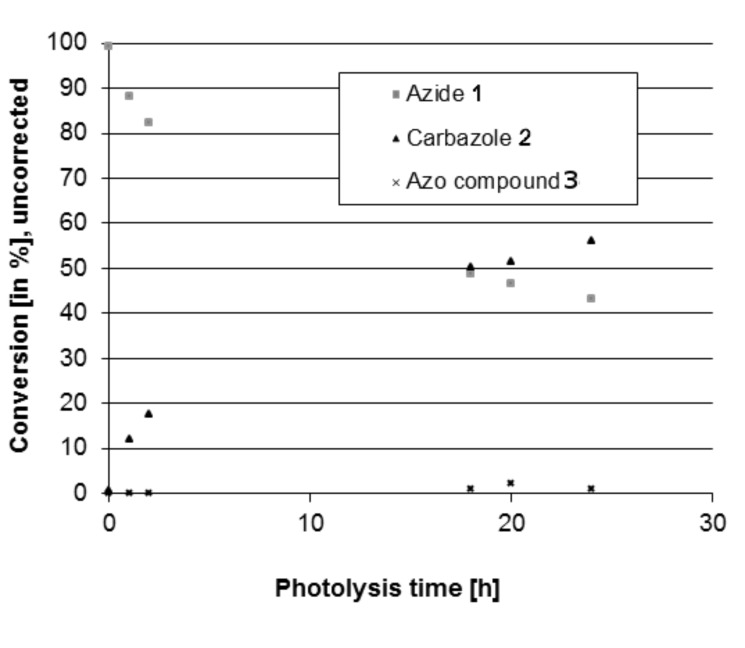
Photolysis results in batch setup (flask) with a xenon lamp (400 W, λ > 345 nm).

We therefore investigated whether the photoreaction can be realized in miniaturized photoreactors and to what extent the use of a laser as a photon source is advantageous. The irradiation of 2-azidobiphenyl (**1**) in methanol with a conventional xenon lamp (400 W, λ > 345 nm) required 18 h for 50% yield (95% selectivity) in a 10 mm cell with an 8 mm light-exposure diameter (Figure 2).

**Figure 3:**
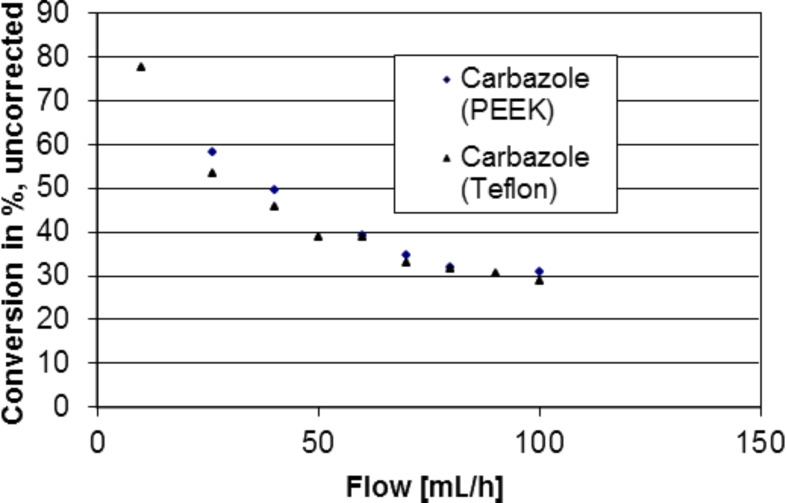
Carbazole synthesis in miniaturized photoreactors Type II (PEEK and Teflon), flow control, P = 0.92 W.

**Figure 4:**
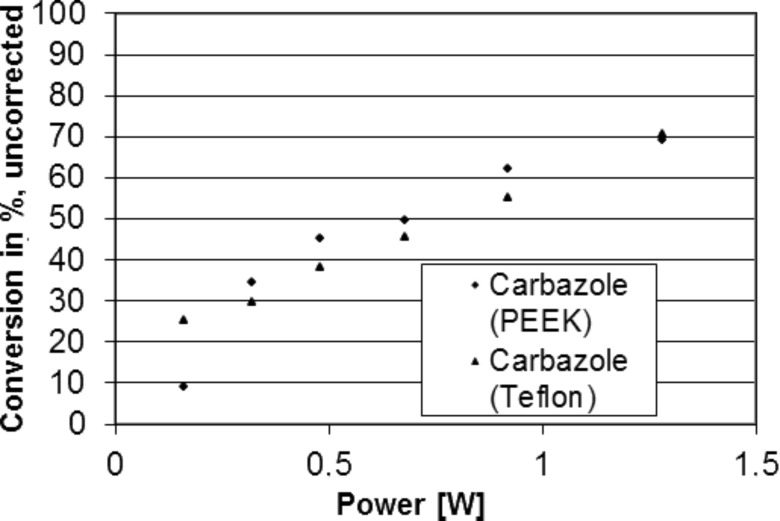
Carbazole synthesis in miniaturized photoreactors Type II (PEEK and Teflon), power control, flow 26 mL/h.

**Figure 5:**
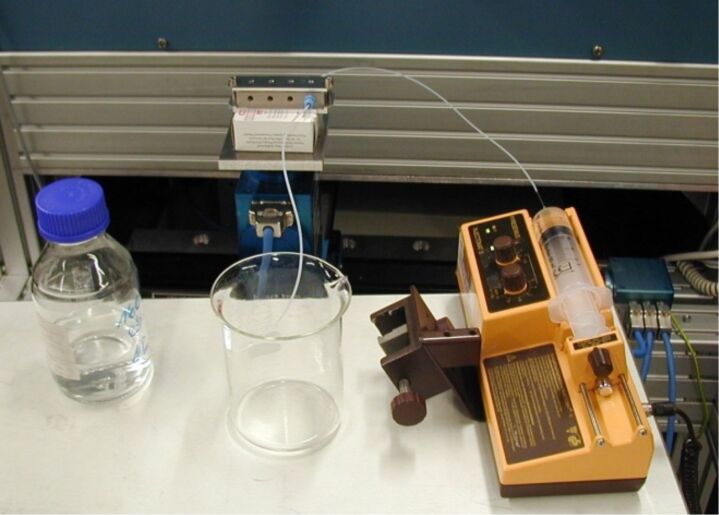
Test setup with continuously operating, miniaturized photoreactor.

**Figure 6:**
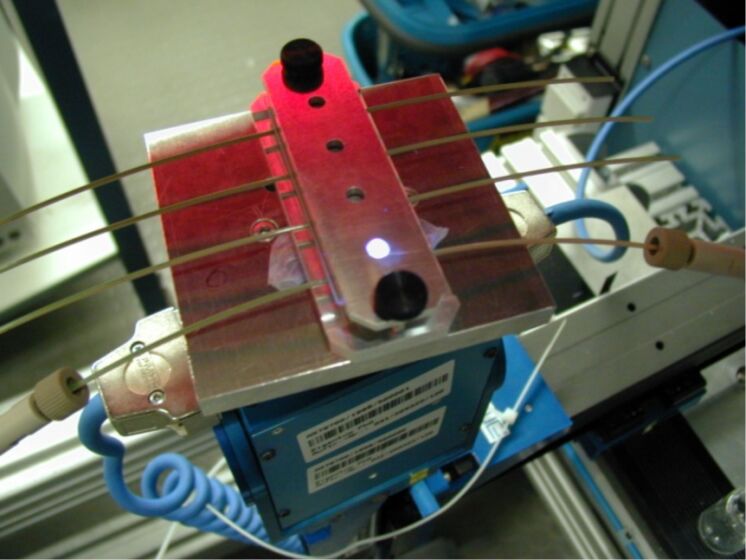
The miniaturized photoreactor (PEEK) during photolysis.

Frequency-tripled Nd:YAG laser radiation (λ = 355 nm, 8 kHz pulse frequency; pulse duration 26 ns) was chosen because the wavelength is close to that of the applied UV-lamp, 355 nm is usually within the absorption area of azides, and this laser type is commonly used in most laser labs. We applied a single-pulse power of 0.16 to 3 W resulting in pulse energies between 4 and 87 nJ and energy densities of approximately 0.02 to 0.17 µJ/cm^2^ within a defocused laser spot of 0.2 to 0.5 cm^2^, to carry out the same reaction (Scheme 1), but carbazole was obtained much faster from 2-azidobiphenyl (**1**). Compared to conventional UV sources, the use of laser irradiation clearly accelerated the reaction: from 18 h (Xe lamp, Figure 2) to 30 s (Nd:YAG laser) for 50% yield and 95% selectivity, calculated from the data presented in Figure 3. This reaction was successfully carried out in a miniaturized photoreactor (Figures 3–6).

The monomolecular reaction can be realized by using laser radiation of 355 nm wavelength as a photon source, in a clean way, avoiding almost completely the formation of the undesired diazo derivatives **3**. The side reaction is supposedly reduced due to a lesser effect of heating owing to the small bandwidth irradiation and minimized exposure time through the miniaturized flow setup.

During these tests, it was shown that a largely better selectivity can be achieved, compared to the one obtained in a standard UV irradiation setup (Figure 2). Experiments were performed in batch as well as flow-injection configuration. The continuous process used allowed us to vary the residence time in the reactor by regulating the flow speed of the reactant solution, with the help of a syringe pump (Figure 5).

For this study, reactors made of PEEK, as well as PTFE reactors were used, leading to similar yields of carbazole (Figure 3 and Figure 4), showing no major influence of the reactor material on the reaction.

The deviations from linearity in the low power area of Figure 4 can be attributed to fluctuations of the laser power. For the high-power area, a correlation between yield, power and reaction time, which can be explained by kinetics, is observed.

## Conclusion

The preparation and application of polymeric, miniaturized photoreactors, equipped for the effective use of photons in the reaction chamber, provided by frequency-converted laser sources, was successfully shown.

With these reactors or reactor components, the photonic influence on reactions in miniaturized photoreactors was proven to be useful in parameter studies in which laser power and flow rate were varied.

The advantages of laser chemistry in the condensed phase compared to standard photochemical approaches have been shown in this preliminary study, proving the suitability of laser photochemistry for organic synthesis. Thanks to the further miniaturization and the availability of new moderately priced laser systems even better suited beam sources can be provided for photochemistry.

In the described experiments, laser radiation of 355 nm wavelength (frequency-tripled Nd:YAG) was used. Since the spectral range of interest for most photoreactions ranges from the ultraviolet to the visible region, tunable laser systems (optical parametric oscillators) feature promising properties for use photochemical experiments. Thus, the irradiation wavelength can be adapted to the needs of the reaction (e.g., to a shifted absorption maximum of the reactant due to substitution) facilitating a large range of applications of this technique. Furthermore, IR laser sources (diode laser, Nd:YAG laser, CO_2_ laser) could be applied for pulsed temperature and pressure elevation in microreactors, as well as microwave stimulation to accelerate reactions.

## Experimental

All starting materials and products were characterized by standard techniques (^1^H NMR, ^13^C NMR, and elemental analysis) and are compared with authentic samples. The products were analyzed by GC–MS (internal standard, dodecane) and/or HPLC.

A solution of 2-azidobiphenyl (**1**) was continuously added to a miniaturized reactor of type II (see Figure 1, dimension of the reactor chamber 3 × 5 mm, 35 μL volume) with a syringe pump. The chamber was continuously irradiated with a Nd:YAG laser (355 nm). At a constant flow of 26 mL/h, the laser-pulse power was varied from 0.16 to 1.28 W. Furthermore, at a constant intermediate power of 0.92 W the flow rate (10 to 100 mL/h) and therefore the dwell time (exposure time) in the reactor was varied. The yield was determined by HPLC.

## Supporting Information

File 1 Description of the flow reactor setup, kinetics, experimental procedures and spectroscopic data of all compounds.
