# Supplementary material for: Photochemistry with laser radiation in condensed phase using miniaturized photoreactors
Source: Beilstein J Org Chem. 2012 Jul 31;8:1213–8. doi: 10.3762/bjoc.8.135 (PMC3458740; doi:10.3762/bjoc.8.135)
Supplement: File 1 — Description of the flow reactor setup, kinetics, experimental procedures and spectroscopic data of all compounds. [file Beilstein_J_Org_Chem-08-1213-s001.pdf]

# Supporting Information

# for

# Photochemistry with laser radiation in condensed phase using miniaturized photoreactors

Elke Bremus-Köbberling[1], Arnold Gillner[1], Frank Avemaria[2], Céline Réthoré[2] and Stefan Bräse*[2,3]

Address: [1]Fraunhofer Institute for Laser Technology, Steinbachstrasse 15, D-52074 Aachen, Germany, [2]Institute of Organic Chemistry, Karlsruhe Institute of Technology, Fritz-Haber-Weg 6, D-76131 Karlsruhe, Germany and [3]Institute of Toxicology and Genetics, Karlsruhe Institute of Technology, Hermann-von-Helmholtz-Platz 1, D-76344 Eggenstein-Leopoldshafen, Germany

Email: Stefan Bräse - braese@kit.edu

* Corresponding author

**Description of the flow reactor setup, kinetics, experimental procedures and spectroscopic data of all compounds.**

## General

NMR spectra were recorded on a Bruker AM 400, a Bruker Avance 300 or a Bruker DRX 500 spectrometer as solutions. Chemical shifts are expressed in parts per million (ppm, $\delta$) downfield from tetramethylsilane (TMS) and are referenced to residual solvent peaks. All coupling constants (*J*) are absolute values and *J* values are expressed in Hertz (Hz). The description of signals include s = singlet, d = doublet, t = triplet, q = quartet, m = multiplet. The spectra were analyzed according to first order. The signal structure in $^{13}$C NMR was analyzed by DEPT and is described as follows: + = primary or tertiary C-atom (positive signal), – = secondary C-atom (negative signal) and $C_{quart}$ = quaternary C-atom (no signal). – EIMS (electron impact mass spectrometry) was performed by using a Finnigan MAT 90 (70 eV). In cases where no MS (EI) spectra could be measured due to the high volatility of the compound, the GC–MS spectra were used for characterization. IR (infrared spectroscopy) was recorded on a FT-IR Bruker alpha. Solvents, reagents and chemicals were purchased from Aldrich, ABCR and Acros. All solvents, reagents and chemicals were used as purchased unless stated otherwise.

## Microreactors used for irradiation

**Synthesis of 2-azidobiphenyl (1) from 2-aminobiphenyl** [1,2]

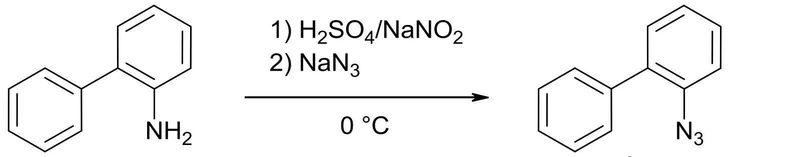


**1**

The amine (5.0 g, 30.1 mmol) was treated with 6 mL of $H_2SO_4$ (concentrated) in 35 mL of $H_2O$. The solution was cooled to 0 °C and 2.48 g of $NaNO_2$ (36 mmol) in 25 mL $H_2O$ was added dropwise. The solution became clear and was treated with sodium azide followed by stirring overnight at rt. After filtration and washing (10% $Na_2CO_3 \cdot H_2O$) the solid was dried.

Yield: 5.35 g (91%)

Analytical data:

| | |
|---|---|
| mp: | 50 °C (Lit.: 49–50 °C) |
| GC: | Ret./min: 13.0 (98.6%) |
| GC–MS: | Ret./min: 13.2 |
| | MS: $M^+$ 195 |
| HPLC: | Ret./min: 2.7 (0.21%), 3.0 (0.49%), 5.1 (99.3%) |

AK Prof. Braese
Name Avemaria
FA-215
AUTO.N.1H_STAND MeOD u braese 3

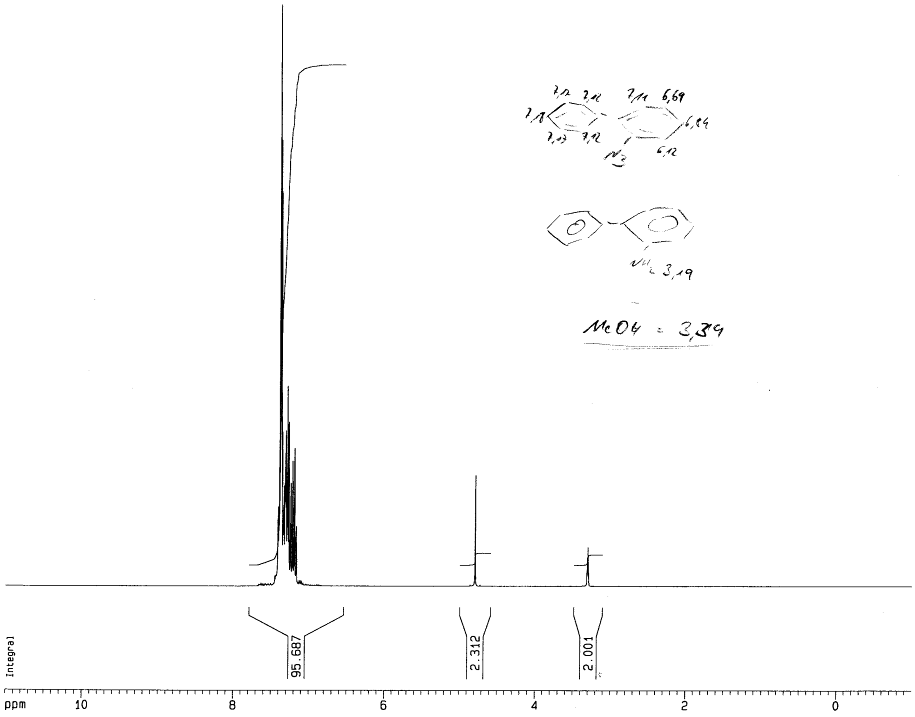


Current Data Parameters
NAME 12x3b003
EXPNO 10
PROCNO 1

F2 - Acquisition Parameters
Date_ 20020323
Time 2.24
INSTRUM spect
PROBHD 5 mm QNP 1H/1
PULPROG zg
TD 65536
SOLVENT MeOD
NS 16
DS 2
SWH 6172.839 Hz
FIDRES 0.094190 Hz
AQ 5.3084660 sec
RG 143.7
DW 81.000 usec
DE 6.00 usec
TE 303.0 K
D1 1.00000000 sec

======== CHANNEL f1 ========
NUC1 1H
P1 11.20 usec
PL1 0.00 dB
SFO1 300.1318534 MHz

F2 - Processing parameters
SI 32768
SF 300.1300075 MHz
WDW EM
SSB 0
LB 0.30 Hz
GB 0
PC 5.00

1D NMR plot parameters
CX 22.00 cm
CY 14.00 cm
F1P 11.000 ppm
F1 3301.43 Hz
F2P -1.000 ppm
F2 -300.13 Hz
PPMCM 0.54545 ppm/cm
HZCM 163.70728 Hz/cm

AK Prof. Braese
Name Avemaria
FA-215
AUTO.N.13C_CPD MeOD u braese 3

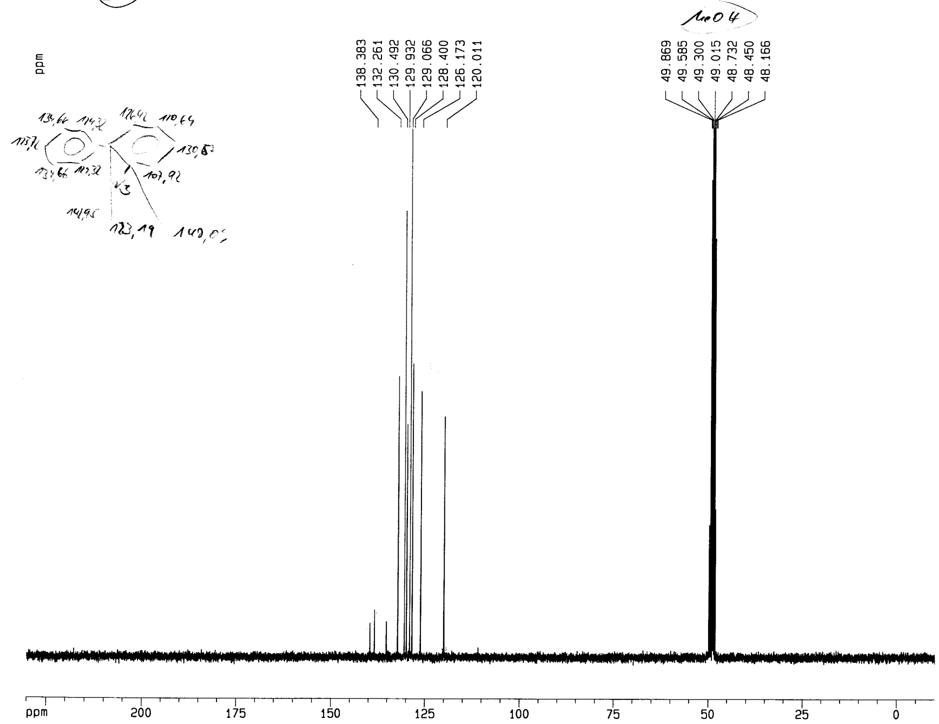


Current Data Parameters
NAME 12x3b003
EXPNO 11
PROCNO 1

F2 - Acquisition Parameters
Date_ 20020323
Time 2.35
INSTRUM spect
PROBHD 5 mm QNP 1H/1
PULPROG zgpg40
TD 65536
SOLVENT MeOD
NS 256
DS 4
SWH 19607.844 Hz
FIDRES 0.299192 Hz
AQ 1.6712180 sec
RG 2896.3
DW 25.500 usec
DE 6.00 usec
TE 303.0 K
D1 0.60000002 sec
d11 0.03000000 sec
d12 0.00002000 sec

======== CHANNEL f1 ========
NUC1 13C
P1 9.80 usec
PL1 3.00 dB
SFO1 75.4760204 MHz

======== CHANNEL f2 ========
CPDPRG2 waltz16
NUC2 1H
PCPD2 108.00 usec
PL2 0.00 dB
PL12 20.00 dB
PL13 26.00 dB
SFO2 300.1314406 MHz

F2 - Processing parameters
SI 32768
SF 75.4676428 MHz
WDW EM
SSB 0
LB 0.60 Hz
GB 0
PC 5.00

1D NMR plot parameters
CX 22.00 cm
CY 12.70 cm
F1P 230.000 ppm
F1 17357.56 Hz
F2P -10.000 ppm
F2 -754.68 Hz
PPMCM 10.90909 ppm/cm
HZCM 823.28339 Hz/cm

**Photolysis of 2-azidobiphenyl in benzene** [2]

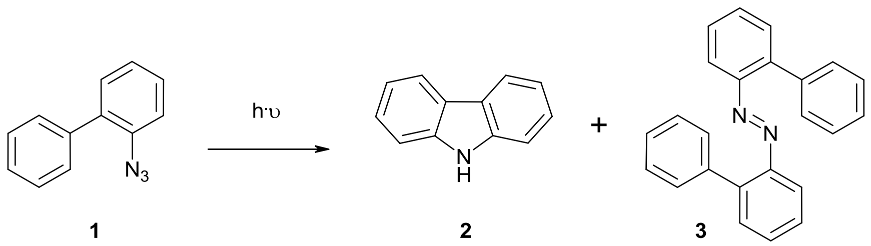

2-Azidobiphenyl (**1**, 0.502 g, 2.58 mmol) was dissolved in 150 mL of degassed benzene under argon ($c$ = 1.73 × $10^{-2}$ M, Lit: 2.0 × $10^{-2}$ M). The reaction solution was heated to 20 °C (cryostat). The photolysis was carried out under stirring and continuous irradiation (xenon lamp, 450 W, λ > 345 nm). The pale yellow solution turned reddish–orange in 20 min and a weak gas evolution was observed. The irradiation took place over 24 h. Samples (1 h, 2 h, 18 h, 24 h) were taken and the turnover was determined by GC. The reaction solution was evaporated and dried over magnesium sulfate to give a dark yellow oil.

Analytical data:

Sample after 1 h:

| | |
|---|---|
| GC: | Ret./min: 1.06 (14.81%), 1.14 (4.36%), 12.93 (70.81%), 14.94 (9.63%) |
| | Ratio starting material:product: 7:1 |

Sample after 2 h:

| | |
|---|---|
| GC: | Ret./min: 1.07 (29.21%), 1.15 (8.49%), 12.92 (50.77%), 14.93 (10.85%) |
| | Ratio starting material:product: 5:1 |

Sample after 18 h:

| | |
|---|---|
| GC: | Ret./min: 1.07 (13.63%), 1.15 (3.96%), 15.0 (39.65%), 15.00 (40.87%) |
| | Ratio starting material:product: 1:1 |

Sample after 24 h:

| | |
|---|---|
| GC: | Ret./min: 1.05 (13.95%), 1.12 (4.06%), 12.92 (34.88%), 14.97 (45.33%) |
| | Ratio starting material:product: 3:4 |
| HPLC: | Ret./min: |
| | 0.53 (2.69%), 1.58 (2.86%), 1.95 (1.56%), 2.91 (57.1%), 5.03 (34.2%) |
| | Ratio starting material:product: 3:5 |

**Chromatographic separation of photolysis reactions.** Eluent: 10% ether in hexane; Column: 35 mm × 500 mm silica gel 60. 1.8 liters of eluent was used, and the collected fractions (41, each ca. 50 mL) by TLC (eluent: 10% ether in hexane) were evaporated.

Fraction 5–9: yellow oil; 2-azobiphenyl:

GC: Ret./min: 13.25 (98.43%)

HPLC: Ret./min: 5.1 (99%)

Fraction 21–37: white crystals; carbazole):

GC: Ret./min: 1.30 (0.25%), 15.05 (99.75%)

HPLC: Ret./min: 3.00 (97.7%)

Elemental analysis:

| Azide **1** 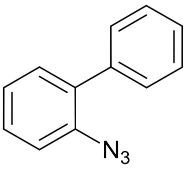 | | | Carbazole **2** 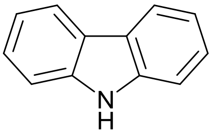 | | |
|---|---|---|---|---|---|
| Calcd. | C | 73.83 | Calcd. | C | 88.88 |
| | H | 4.65 | | H | 2.49 |
| | N | 21.52 | | N | 8.64 |
| Found | C | 73.62 | Found | C | 86.28 |
| | H | 4.02 | | H | 5.83 |
| | N | 21.07 | | N | 8.26 |

**Photolysis of 2-azidobiphenyl (1) in MeOH**

Procedure, see above.

Analysis:

Starting material, azide

GC: Ret./min: 1.23 (0.2%), 11.39 (0.3%), 13.01 (98.3%), 13.84 (0.5%) 14.94 (0.6%)

Sample after 19 h:

GC: Ret./min: 1.29 (0.5%), 11.39 (0.2%), 12.26 (1.0%), 12.95 (45.5%), 15.00 (50.3%), 24.53 (2.1%)

Ratio starting material:product: 10:9

**Graphical representation: conversion of macroscopic experimental procedure of photolysis, a conventional reaction flask, Xe-lamp**

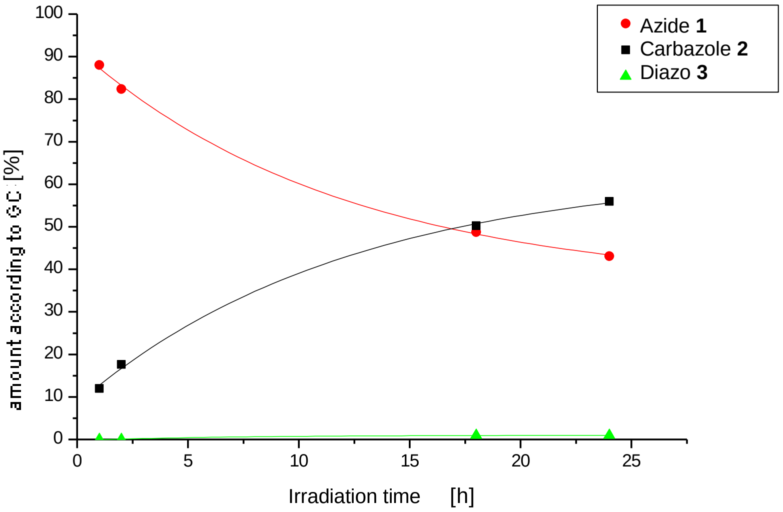


• Products were formed in a first-order kinetic during irradiation

• Plot shows conversion in benzene (GC) was slightly better than in MeOH

• Max. conversion at about 51% after 20 h in methanol with 2.22% side-product (diazobenzene)

**Exemplary GC in MeOH:**

Starting material, azide **1**

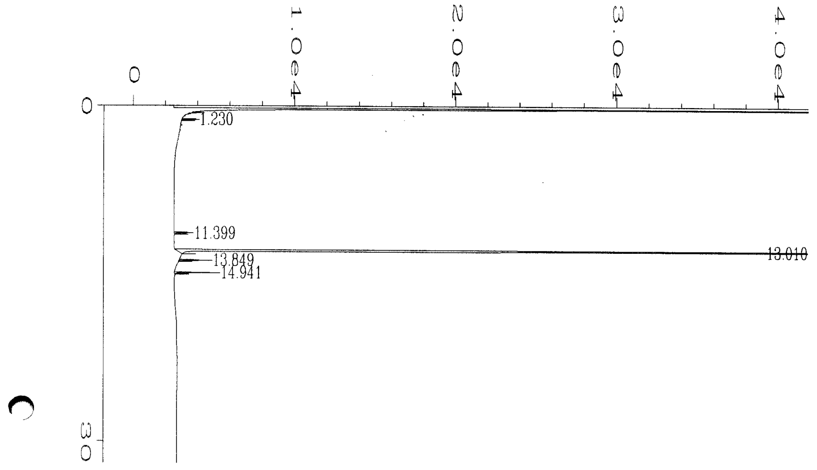


```
=============================================================================
                          Area Percent Report
=============================================================================

Data File Name    : C:\HPCHEM\1\DATA\FA\MP007ED.D
Operator          : FA
Instrument        : INSTRUMEN                       Page Number       : 1
Sample Name       : mp007ed                         Vial Number       : 1
Run Time Bar Code:                                  Injection Number :
Acquired on       : 11 Jul 98   11:17 AM            Sequence Line     :
Report Created on: 11 Jul 98   11:50 AM             Instrument Method: DR40.MTH
                                                    Analysis Method   : DR40.MTH
```

Sample after 19 h

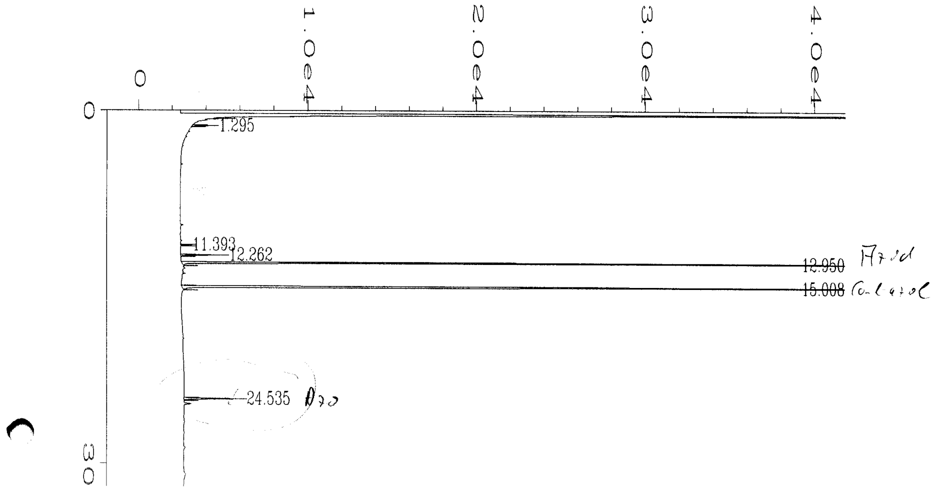


```
=============================================================================
                          Area Percent Report
=============================================================================

Data File Name    : C:\HPCHEM\1\DATA\MP\MP007.D
Operator          : mel
Instrument        : INSTRUMEN                       Page Number       : 1
Sample Name       : mp 007                          Vial Number       : 1
Run Time Bar Code:                                  Injection Number :
Acquired on       : 12 Jul 98   01:59 PM            Sequence Line     :
Report Created on: 12 Jul 98   02:31 PM             Instrument Method: DR40.MTH
                                                    Analysis Method   : DR40.MTH
```

**Photolytic carbazole formation of 2-azobiphenyl using frequency-tripled Nd:YAG laser radiation in a cuvette**

In preliminary experiments solutions of 2-azidobiphenyl were irradiated in quartz-glass cuvettes with a laser source. The power and the irradiation time were varied. Laser source: Nd:YAG laser (355 nm) 355-5000, 210S Lightwave Electronics, USA. The pulse frequency of the laser was fixed at 8 kHz (pulse width: 26 ns). The circular beam diameter on the cuvette (35 × 10 × 10 mm) is around 8 mm. The penetration depth of the laser in the media (cross-section-considered diffusion cone) is estimated to be about 7 mm. The irradiation time by means of repeated scanning of the specimen varies. It was proceeded at a speed $v$ = 10 mm $s^{-1}$ with a cell length of 30 mm, resulting in an irradiation time per scan of 3 s. The pulse power of the laser was in the range of 0.5 to 3 W corresponding to energy densities of 0.03 to 0.17 µJ/$cm^2$, and the irradiation time in 2–80 scans (equivalent to 6–240 s / 4 min, see table below).

| Power P [Watt] | Time # Scans | Time *t* [sec] | Carbazole (HPLC, %) | Azide (HPLC, %) | Product/Educt (HPLC) | Conversion in % (HPLC) |
|---|---|---|---|---|---|---|
| 0,50 | 20 | 60 | 46,5 | 52,5 | 0,89 | 0,009 |
| 1,50 | 1 | 3 | 12,6 | 87,3 | 0,14 | 0,001 |
| 1,50 | 10 | 30 | 52,4 | 46,5 | 1,13 | 0,011 |
| 1,50 | 20 | 60 | 56,4 | 42,1 | 1,34 | 0,013 |
| 1,50 | 20 | 60 | 62,5 | 35,5 | 1,76 | 0,018 |
| 1,50 | 20 | 60 | 58,8 | 39,3 | 1,50 | 0,015 |
| 1,50 | 40 | 120 | 76,0 | 21,5 | 3,53 | 0,035 |
| 1,50 | 80 | 240 | 90,3 | 5,8 | 15,70 | 0,157 |
| 3,00 | 20 | 60 | 73,7 | 23,9 | 3,08 | 0,031 |
| 3,00 | 20 | 60 | 79,8 | 17,4 | 4,58 | 0,046 |
| 3,00 | 40 | 120 | 87,3 | 9,1 | 9,60 | 0,096 |
| 3,00 | 80 | 240 | 92,8 | 1,5 | 60,23 | 0,602 |

**Graphical representation: Conversion of the photolysis laser radiation (355 nm) in a cuvette**

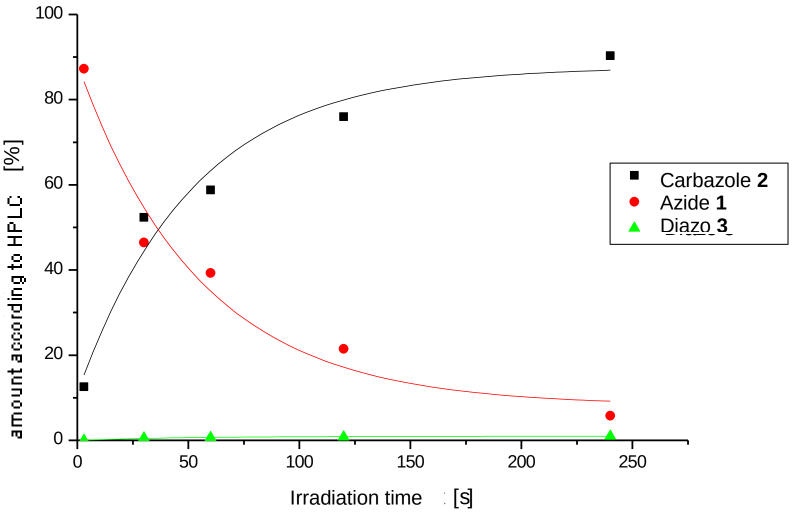


• Products of the exponential growth with time

• Max. turnover at about 90% after 4 min in methanol with 0.95% side-product (diazobenzene)

• Conversion of 52% and 0.57% side product (diazobenzene, after 30 s)

.

Exemplary HPLC

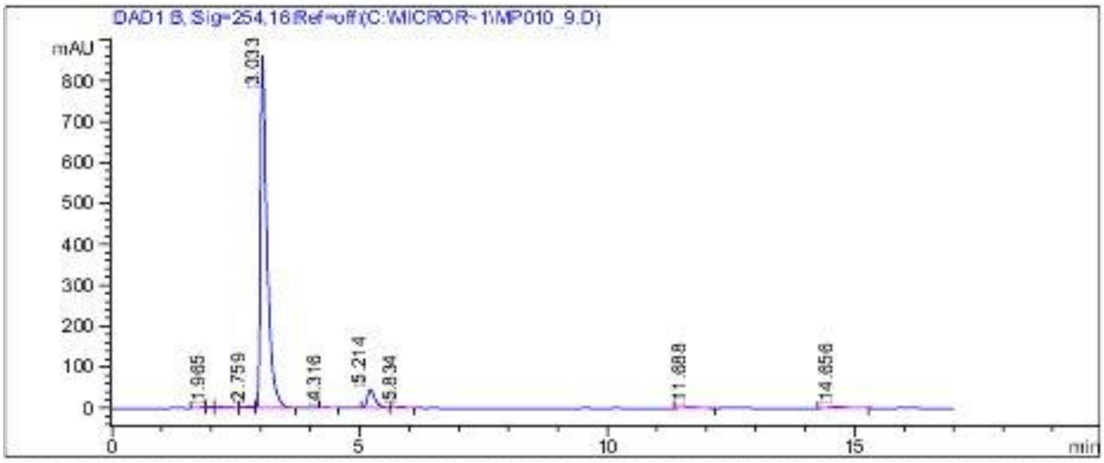


| Peak # | RetTime [min] | Type | Width [min] | Height [mAU] | Height % | Area [mAU*s] | Area % |
|---|---|---|---|---|---|---|---|
| 1 | 1.832 | PV | 0.111 | 2.73949 | 0.29402 | 21.3533 | 0.25 |
| 2 | 1.965 | VV | 0.129 | 3.30173 | 0.35436 | 29.7444 | 0.34 |
| 3 | 2.173 | VV | 0.247 | 2.90165 | 0.31142 | 53.4776 | 0.62 |
| 4 | 2.759 | VV | 0.152 | 5.10301 | 0.54769 | 57.0789 | 0.66 |
| 5 | 3.033 | VP | 0.128 | 862.00085 | 92.51538 | 7798.4937 | 90.30 |
| 6 | 4.316 | PB | 0.137 | 1.49874 | 0.16085 | 14.0396 | 0.16 |
| 7 | 5.214 | BB | 0.159 | 45.49478 | 4.88279 | 496.2671 | 5.75 |
| 8 | 5.834 | BB | 0.196 | 2.98286 | 0.32014 | 40.3533 | 0.47 |
| 9 | 11.688 | PP | 0.296 | 2.27184 | 0.24383 | 44.0054 | 0.51 |
| 10 | 14.656 | BB | 0.369 | 3.44291 | 0.36951 | 81.6693 | 0.95 |
| Totals : | | | | 931.73786 | | 8636.4825 | |

**Photolytic carbazole formation of 2-azobiphenyl using frequency-tripled Nd:YAG laser radiation in a continuously operating microreactor (PTFE)**

In miniaturized reactors of type II (see Figure 1, 3 and 4 of the manuscript, dimension of the reactor chamber 3 × 5 mm, 35 μL volume) was added a solution of 2-azidobiphenyl continuously fed with a syringe pump under continuous irradiation with a Nd:YAG laser (355 nm irradiated). At a constant flow of 26 mL/h, the laser pulse power was varied from 0.16 to 1.28 W corresponding to energy densities between 0.02 and 0.17 μJ/cm$^2$. Furthermore, at a constant power of 0.92 W or 0.12 μJ/cm$^2$, the flow rate (10 to 100 mL/h) and therefore the dwell time (exposure time) in the reactor were varied. The yield was determined by HPLC.

**HPLC determination of conversion by the photolysis laser radiation (355 nm, constant flow rate 26 mL/h, power variable) in the continuously operated microreactor (PTFE)**

| Pulse power [W] | Carbazole | Azide |
|---|---|---|
| 0.16 | 25.23 | 73.57 |
| 0.32 | 29.91 | 68.78 |
| 0.48 | 38.13 | 60.29 |
| 0.68 | 45.59 | 52.61 |
| 0.92 | 55.28 | 42.44 |
| 1.28 | 70.80 | 26.68 |

**Graphical representation: Photolysis in the continuously operated microreactor (PTFE)**

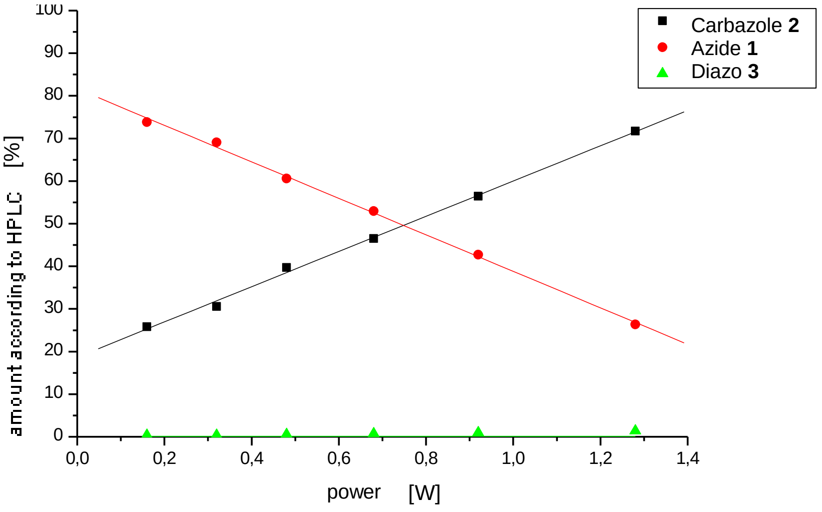


• Products with a linear increase in performance

**HPLC determination of conversion by the photolysis laser radiation (355 nm, 0.92 W constant power, variable flow rate) in the continuously operated microreactor (PTFE)**

| Carbazole [%, HPLC] | Azide [%, HPLC] | Time [s] | Flow [mL/h] |
|---|---|---|---|
| 77.53 | 19.32 | 12.3 | 10 |
| 53.25 | 44.53 | 4.73 | 26 |
| 45.86 | 52.31 | 3.07 | 40 |
| 38.69 | 59.70 | 2.46 | 50 |
| 38.90 | 59.50 | 2.05 | 60 |
| 33.00 | 65.57 | 1.76 | 70 |
| 31.59 | 67.02 | 1.54 | 80 |
| 30.46 | 68.19 | 1.37 | 90 |
| 28.73 | 69.98 | 1.23 | 100 |

**Graphical representation: Conversion of the photolysis laser radiation (355 nm) in the continuously operated microreactor (PTFE)**

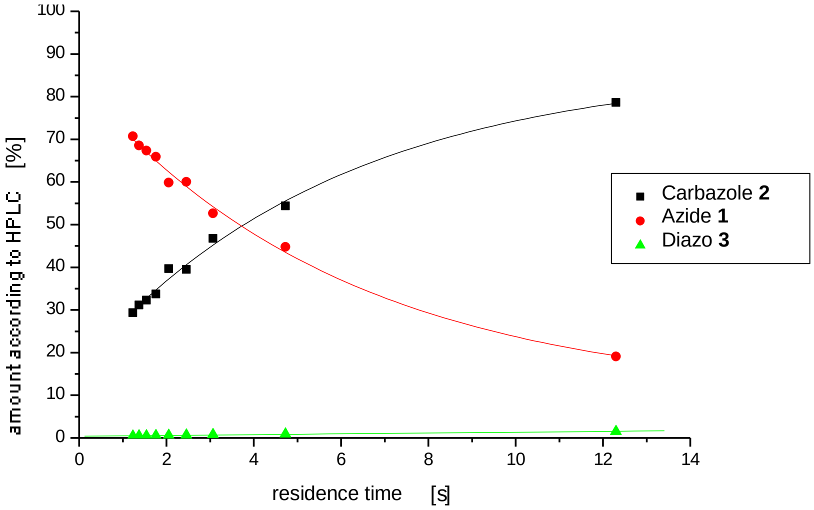


- Products with an exponential increase of the time
- Max. conversion at about 79% at a residence in the reaction solution in the miniaturized photoreactor of 12.3 seconds at 1.52% side-product (diazobenzene)

# References:


1. Smith, Brown; *J. Am. Chem. Soc. 1951*, *73,* 2438–2441
2. Swenton, J. S.; Ikeler, T. J.; Williams, B.H. *J. Am. Chem. Soc.* **1970**, *92*, 3103–3109
